# Short-Term Colony-Stimulating Factor 1 Receptor Inhibition–Induced Repopulation After Stroke Assessed by Longitudinal ^18^F-DPA-714 PET Imaging

**DOI:** 10.2967/jnumed.121.263004

**Published:** 2022-09

**Authors:** Cristina Barca, Amanda J. Kiliaan, Lydia Wachsmuth, Claudia Foray, Sven Hermann, Cornelius Faber, Michael Schäfers, Maximilian Wiesmann, Bastian Zinnhardt, Andreas H. Jacobs

**Affiliations:** 1 European Institute for Molecular Imaging, University of Münster, Münster, Germany;; 2 Department of Medical Imaging/Anatomy, Radboud University Medical Center, Radboud, The Netherlands;; 3 Translational Research Imaging Center, University Hospital Münster, Münster, Germany;; 4 Department of Nuclear Medicine, University Hospital Münster, Münster, Germany;; 5 Biomarkers and Translational Technologies, Pharma Research and Early Development, F. Hoffmann-La Roche Ltd., Basel, Switzerland; and; 6 Department of Geriatrics and Neurology, Johanniter Hospital, Bonn, Germany

**Keywords:** colony-stimulating factor 1 receptor, microglia, stroke, ^18^F-DPA-714, repopulation

## Abstract

Studies on colony-stimulating factor 1 receptor (CSF-1R) inhibition–induced microglia depletion indicated that inhibitor withdrawal allowed the renewal of the microglia compartment via repopulation and resolved the inflammatory imbalance. Therefore, we investigated for the first time (to our knowledge) the effects of microglia repopulation on inflammation and functional outcomes in an ischemic mouse model using translocator protein (TSPO)-PET/CT and MR imaging, ex vivo characterization, and behavioral tests. **Methods:** Eight C57BL/6 mice per group underwent a 30-min transient occlusion of the middle cerebral artery. The treatment group received CSF-1R inhibitor in 1,200 ppm PLX5622 chow (Plexxikon Inc.) from days 3 to 7 to induce microglia/macrophage depletion and then went back to a control diet to allow repopulation. The mice underwent T2-weighted MRI on day 1 after ischemia and ^18^F-labeled *N,N*-diethyl-2-(2-[4-(2-fluoroethoxy)phenyl]-5,7-dimethylpyrazolo[1,5-α]pyrimidine-3-yl)acetamide (^18^F-DPA-714) (TSPO) PET/CT on days 7, 14, 21, and 30. The percentage injected tracer dose per milliliter within the infarct, contralateral striatum, and spleen was assessed. Behavioral tests were performed to assess motor function recovery. Brains were harvested on days 14 and 35 after ischemia for ex vivo analyses (immunoreactivity and real-time quantitative polymerase chain reaction) of microglia- and macrophage-related markers. **Results:** Repopulation significantly increased ^18^F-DPA-714 uptake within the infarct on days 14 (*P* < 0.001) and 21 (*P* = 0.002) after ischemia. On day 14, the ionized calcium binding adaptor molecule 1 (Iba-1)–positive cell population showed significantly higher expression of TSPO, CSF-1R, and CD68, in line with microglia repopulation. Gene expression analyses on day 14 indicated a significant increase in microglia-related markers (*csf-1r, aif1,* and *p2ry12*) with repopulation, whereas peripheral cell recruitment–related gene expression decreased (*cx3cr1* and *ccr2*), indicative of peripheral recruitment during CSF-1R inhibition. Similarly, uncorrected spleen uptake was significantly higher on day 7 after ischemia with treatment (*P* = 0.001) and decreased after drug withdrawal. PLX5622-treated mice walked a longer distance (*P* < 0.001) and more quickly (*P* = 0.009), and showed greater forelimb strength (*P* < 0.001), than control mice on day 14. **Conclusion:** This study highlighted the potential of ^18^F-DPA-714 PET/CT imaging to track microglia and macrophage repopulation after short-term CSF-1R inhibition in stroke.

## Visual Abstract

**Figure:**
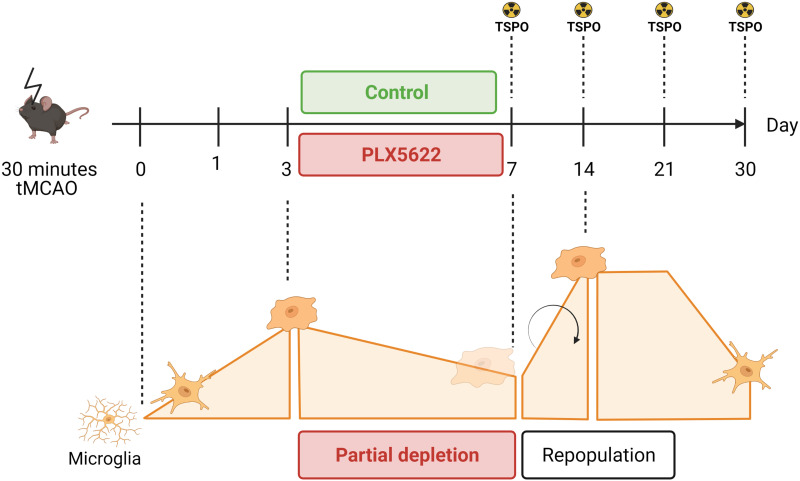

Microglia play a major role in the stroke-induced neuroinflammatory response, as part of the early proinflammatory and later restorative processes (*1*). Microglia survival and proliferation are dependent on signaling through the colony-stimulating factor 1 receptor (CSF-1R) (*2*). Administration of the CSF-1R inhibitor PLX5622 progressively leads to almost complete microglia depletion after 1 wk of treatment in wild-type mice (*3*). Microglia depletion in acute or subacute ischemia (1–3 d) was associated with increased immune cell infiltration and aggravated brain inflammation (*4**,**5*). Inhibitor withdrawal triggers microglia repopulation, indicated by microglia proliferation and increased activity. Besides, long-lasting treatment effects were observed on other cell populations (*6*). Recently, we demonstrated that CSF-1R inhibition–induced microglia/macrophage depletion could be tracked using an ^18^F-labeled radiotracer—*N,N*-diethyl-2-(2-[4-(2-fluoroethoxy)phenyl]-5,7-dimethylpyrazolo[1,5-α]pyrimidine-3-yl)acetamide (^18^F-DPA-714)—targeting the translocator protein (TSPO) (*7*): TSPO-dependent neuroinflammation was significantly decreased within the first weeks after stroke, although long-term CSF-1R inhibition was associated with a poor disease outcome. Therefore, PLX5622 represents an attractive microglia- and macrophage-targeting pharmacologic tool allowing modulation of the inflammatory environment after stroke. However, microglia repopulation has yet to be investigated.

The therapeutic effect of short-term CSF-1R inhibition has scarcely been investigated but has shown promising applications. In a neuronal injury mouse model, renewal of the microglia compartment after short-term CSF-1R inhibition reduced lesion-induced inflammatory markers, resolved the active phenotype of microglia, and reversed behavioral impairment (*3*). Therefore, short-term PLX5622 treatment represents an opportunity to renew the cellular compartment and reprogram microglia activity to reduce ischemia-associated inflammation.

In this study, we aimed to longitudinally investigate the effects of microglia repopulation in a stroke mouse model. We investigated the therapeutic effect of microglia repopulation on stroke outcomes by inhibiting CSF-1R between days 3 and 7 after ischemia. Few studies in stroke models have investigated how microglia affect outcomes after CSF-1R inhibition (*4**,**5**,**8*). Results indicated that absence of microglia within the first days after stroke worsened disease outcomes, including increased brain injury, enhanced excitotoxicity, and brain inflammation. Therefore, we aimed to leverage the phagocytic activity on infiltrating immune cells and cell debris within the first days after ischemia (*4*), to reduce microglia-related excitotoxicity, to renew the microglia compartment, and to take advantage of the repopulation process on lesion-associated inflammation at the peak of TSPO-dependent inflammation reported between days 10 and 14 after ischemia (*9*–*11*).

To investigate the kinetics of immune cell activation, we performed longitudinal PET using the TSPO radiotracer ^18^F-DPA-714 as a marker for neuroinflammation, together with CT and MRI. Imaging data were cross-correlated with protein and gene expression profiles of microglia, macrophages and inflammation-related markers using immunohistochemistry and real-time quantitative polymerase chain reaction. In addition, we studied the therapeutic effect on motor functions using a set of behavioral tests.

We hypothesized that subacute PLX5622 treatment may induce immunomodulatory effects by triggering microglia repopulation, ultimately showing a positive effect on stroke outcomes. The repopulation process, indicated by an increased TSPO PET signal, may be detected noninvasively by ^18^F-DPA-714 PET/MRI.

## MATERIALS AND METHODS

### Study Approval

All experiments were conducted in accordance with the German Law on the Care and Use of Laboratory Animals and approved by the Landesamt für Natur, Umwelt, und Verbraucherschutz of North Rhine–Westphalia and according to the ARRIVE 2.0 guidelines (Animal Research: Reporting of In Vivo Experiments; https://www.nc3rs.org.uk/arrive-guidelines).

### Study

**FIGURE 1.**
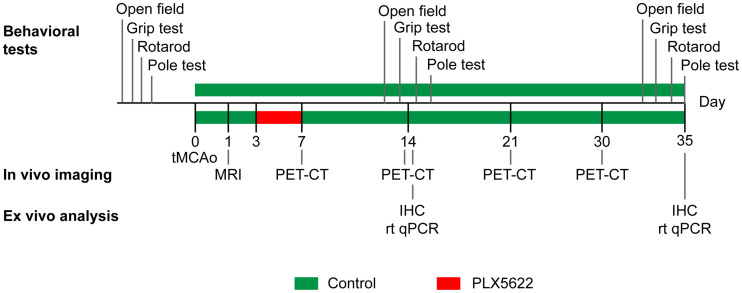
Study design. IHC = immunohistochemistry; rt qPCR = real-time quantitative polymerase chain reaction; tMCAo = transient middle cerebral artery occlusion.

Male C57BL6/J mice 3–4 mo old (*n* = 32) were provided by the local animal facility. They were housed under a standard 12 h:12 h light:dark cycle with free access to food and water. All mice underwent a 30-min transient occlusion of the middle cerebral artery (day 0) and were randomized into either a control group or a PLX5622-treated group by an external person. All mice underwent T2-weighted MRI on day 1. The treatment group received 1,200 ppm PLX5622 chow from days 3 to 7 after ischemia (Fig. 1). The experimenters did not know the group assignment.

Exclusion criteria were lack of reperfusion (<50% baseline cerebral blood flow recovery) as assessed by laser Doppler, an infarct exceeding the striatal and cortical regions, and extreme weight loss (>20% of the initial body weight). The dropout rate was 8%.

Eight animals per group were used for in vivo PET imaging on days 7, 14, 21, and 30 after ischemia. The same animals were used for behavioral assessment before and after surgery. They were killed on day 35 after ischemia to obtain late invasive data. An extra group of 8 animals was added to characterize the 14-d time point as indicated in Supplemental Table 1 (supplemental materials are available at http://jnm.snmjournals.org).

On the last day of the experiment, the mice were killed by transcardial perfusion with phosphate-buffered saline. The brains that were reserved for immunoreactivity were fixed with 4% paraformaldehyde in phosphate-buffered saline, whereas the others, which were kept for real-time quantitative polymerase chain reaction, were flash-frozen in nitrogen and stored at −80°C.

### Surgery

Stroke was induced by the intraluminal suture method as previously described (*12*). Briefly, the mice were anesthetized with 5% isoflurane/O_2_ (Forene; Abbott) after intraperitoneal injection of buprenorphine (0.03 mg/kg of body weight) and maintained at 2.5% v/v isoflurane/O_2_ on a heating pad during the entire procedure.

A 7–0 silicon rubber-coated monofilament (diameter with coating, 0.19 ± 0.01 mm) (Doccol Corp.) was inserted into the right internal carotid artery to block the middle cerebral artery origin. After 30 min, the monofilament was removed to allow reperfusion. The mice received buprenorphine (0.1 mg/kg body of weight) and were placed under the infrared heating lamp until full recovery. Successful occlusion and reperfusion of the middle cerebral artery were assessed by measuring the cerebral blood flow by laser Doppler flowmetry (Periflux 5000; Perimed).

### Treatment

PLX5622 was provided by Plexxikon Inc., formulated in AIN-76A standard chow by Research Diets Inc. at 1,200 ppm and stored at 4°C until use. Both control and PLX5622-enriched diets were provided ad libitum as described in Figure 1. Body weight was reported as an index of food intake (Supplemental Fig. 1).

### ^18^F-DPA-714 PET/CT Imaging

^18^F-DPA-714 (TSPO) PET imaging was performed on days 7, 14, 21, and 30 after ischemia using a high-resolution small-animal PET scanner (32-module quadHIDAC; Oxford Positron Systems Ltd.) with a uniform spatial resolution of less than 1 mm (in full width at half maximum) over a cylindric field of view (165-mm diameter, 280-mm axial length) (*13*).

Radiotracer was prepared as previously described with more than a 99% radiochemical purity (*14*). Once anesthetized, the mice received 12.9 ± 2.2 MBq via the tail vein (specific activity, 40–80 GBq/μmol). The animals were kept anesthetized in a warmed environment for 45 min. The scan was acquired from 45 to 65 min after injection. PET data were reconstructed using a 1-pass list-mode expectation-maximization algorithm with resolution recovery (*13*). The images were corrected only for activity decay. After the PET scan, the animal bed was transferred into a CT scanner (Inveon; Siemens Medical Solutions) with a spatial resolution of 86 μm. The PET/CT images were coregistered using a landmark approach.

### MRI

T2-weighted MRI was performed on day 1 to delineate the infarct. After anesthesia (5% isoflurane/air, Forene; Abbott), the mice were positioned on a heated MRI cradle and fixed by bite and ear bars. The animals were continuously supplied with 2% isoflurane/air until the end of the experiment. Respiration and body temperature (37.0°C ± 0.5°C) were constantly monitored. A small sheet prepared from 1% v/v agar/water solution was placed directly on the animal’s head to reduce susceptibility artifacts and covered with parafilm (Merck KGaA). Two different systems were used.

With the first system, the cradle was manually positioned in the center of the 9.4-T small-animal MRI scanner (Biospec 94/20; Bruker Biospin GmbH). All images were processed and generated using Paravision 5.1 (Bruker Biospin MRI). T2-weighted images were acquired with a fast spin-echo sequence (rapid acquisition with relaxation enhancement) (repetition time, 7,700 ms; effective echo time, 100 ms; rapid-acquisition-with-relaxation-enhancement factor, 30; field of view, 2 × 2 cm; slice thickness, 0.5 mm; interslice distance, 0 cm; number of slices, 20; matrix, 192 × 192; number of averages, 8).

With the second system, a T2-weighted fast spin-echo 2-dimensional sequence was acquired in a 1-T nanoScan PET/MRI scanner equipped with an MH20 coil (resolution, 0.27 × 0.27 × 0.9 mm; Mediso Medical Imaging Systems).

### Behavioral Tests

Open-field (OF), grip, rotarod, and pole testing was performed to assess the treatment effect on motor function recovery as previously described (*7**,**15*). The 4 behavioral tests were performed the week before surgery and on days 14 and 35, as indicated in Figure 1. The protocols are described in the supplemental materials and methods.

### Image Analysis

PET/CT images from the same mouse were manually coregistered with T2-weighted MR images acquired on day 1. An atlas was adjusted to anatomic landmarks, following bone structures and ventricles, and manually corrected. The infarct was delineated using a thresholding approach previously described (*11*). Uptake was assessed within the T2-weighted MRI-defined infarct, the atlas-based contralateral striatum, and manually delineated spleen. Uptake was reported as percentage injected dose per milliliter in both regions, and the infarct-to-contralateral striatum ratio was calculated.

### Immunoreactivity

Brains were fixed in 4% paraformaldehyde, embedded in paraffin, and cut into 5-μm coronal sections. Immunohistochemistry and immunofluorescence were performed as previously described (*7*). Ionized calcium binding adaptor molecule 1 (Iba-1), glial fibrillary acidic protein (GFAP), and TSPO expression level were visualized and quantified by immunoreactivity. Moreover, the TSPO cellular source was assessed with Iba-1/TSPO, CSF-1R/TSPO, CD68/TSPO, and GFAP/TSPO costaining. Primary and secondary antibodies are reported in Supplemental Table 2.

Images were obtained using a confocal microscope (Eclipse NI-E; Nikon) and displayed with ImageJ software.

### Real-Time Quantitative Polymerase Chain Reaction

Real-time quantitative polymerase chain reaction was performed from snap-frozen half-brain tissues as previously described (*7*). The forward- and reverse-primer sequences are reported in Supplemental Table 3. Relative gene expression was assessed using the ΔΔCt method, with Gapdh (Biomol Gmbh) as a housekeeping gene.

### Statistical Analysis

All statistical analyses were performed using SigmaPlot, version 13.0 (Systat Software). The datasets were tested for normal distribution and equal variance.

The sample size was based on effect size (*P* = 0.05; power, 1-β = 0.80), mortality rates, and previous stroke studies (*7**,**15*), in which we investigated the therapeutic effect of dietary approaches on brain inflammation as assessed by ^18^F-DPA-714 PET imaging. Intraindividual ^18^F-DPA-714 PET imaging data, behavioral testing, gene expression, and immunoreactivity datasets were analyzed by repeated-measures ANOVA followed by the Sidak post hoc test for multiple comparisons, unless stated otherwise. All data are expressed as mean ± SEM.

## RESULTS

Both experimental groups showed a similar infarct size on day 1 (*P* = 0.59) (Supplemental Fig. 2).

**FIGURE 2.**
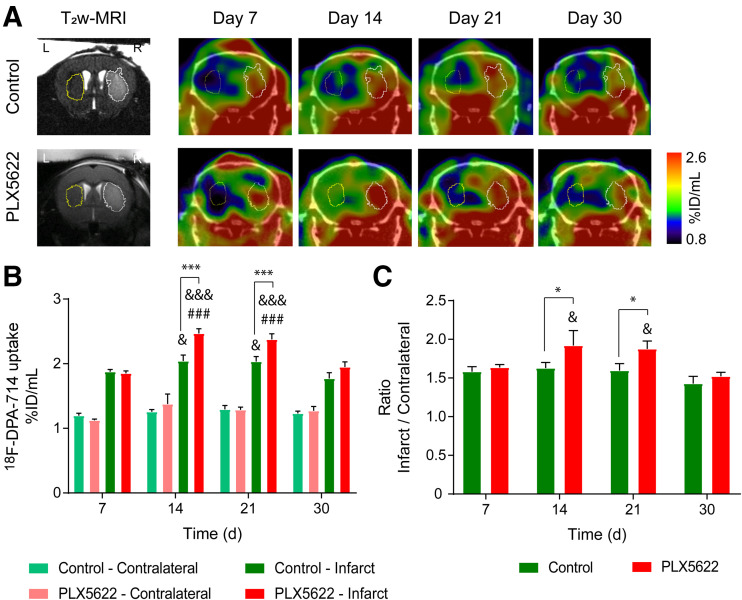
(A) Representative ^18^F-DPA-714 PET/CT images and corresponding T2-weighted MR images. (B) Quantification of mean ^18^F-DPA-714 uptake (percentage injected dose/mL) within infarct and contralateral striatum. (C) Infarct-to-contralateral striatum ratio. Treatment: ***P < 0.005. Day 7: ^###^*P* < 0.005. Day 30: ^&^*P* < 0.05, ^&&&^*P* < 0.005. %ID = percentage injected dose; T_2_w = T2-weighted.

We performed longitudinal ^18^F-DPA-714 PET/CT imaging to assess the repopulation process (Fig. 2A). ANOVA indicated significantly increased uptake within the infarct compared with the contralateral striatum over time (Supplemental Fig. 3). Two-way repeated-measures ANOVA indicated a significant effect of time (*P* < 0.001), treatment (*P* = 0.007), and time × treatment (*P* = 0.009) on uptake within the infarct (Fig. 2B). In the control group, intraindividual comparison indicated that the mean uptake decreased on day 30 compared with days 14 (*P* = 0.024) and 21 (*P* = 0.022). In PLX5622-treated mice, uptake increased from day 7 to day 14 (*P* < 0.001) and remained elevated on day 21 (*P* < 0.001). Later, uptake significantly decreased on day 30 compared with days 14 (*P* < 0.001) and 21 (*P* < 0.001) after ischemia. A treatment effect was observed on days 14 and 21 after ischemia: PLX5622-treated mice showed increased ^18^F-DPA-714 uptake within the infarct on both day 14 (*P* < 0.001) and day 21 (*P* = 0.002) compared with control mice. Similarly, the infarct-to-contralateral striatum ratio indicated a significant effect of both time (*P* = 0.008) and treatment (*P* = 0.043) (Fig. 2C). Treatment effects were observed on days 14 and 21: PLX5622-treated mice showed higher mean ratios than did control mice on both day 14 (*P* = 0.043) and day 21 (*P* = 0.041). ^18^F-DPA-714 PET imaging data on days 14 and 30 were further cross-validated by immunohistochemistry (Supplemental Fig. 4).

The temporal dynamic of ^18^F-DPA-714 uptake within the spleen indicated a treatment effect (*P* = 0.027) on days 7 and 30 after ischemia, with PLX5622-treated mice showing higher uptake than control mice (Supplemental Fig. 5).

We determined the number of Iba-1–positive cells (microglia and macrophages) within the infarct, at the periphery of the infarct, and in the contralateral striatum for both experimental groups on both day 14 and day 35 (Supplemental Fig. 6). On day 14, qualitative assessment of Iba-1–positive cell morphology indicated a greater ramification in control than in PLX5622-treated mice at both the periphery and the contralateral side. On day 35, PLX5622-treated mice showed a significantly decreased percentage of Iba-1–positive area within the infarct compared with control mice (*P* < 0.001).

**FIGURE 3.**
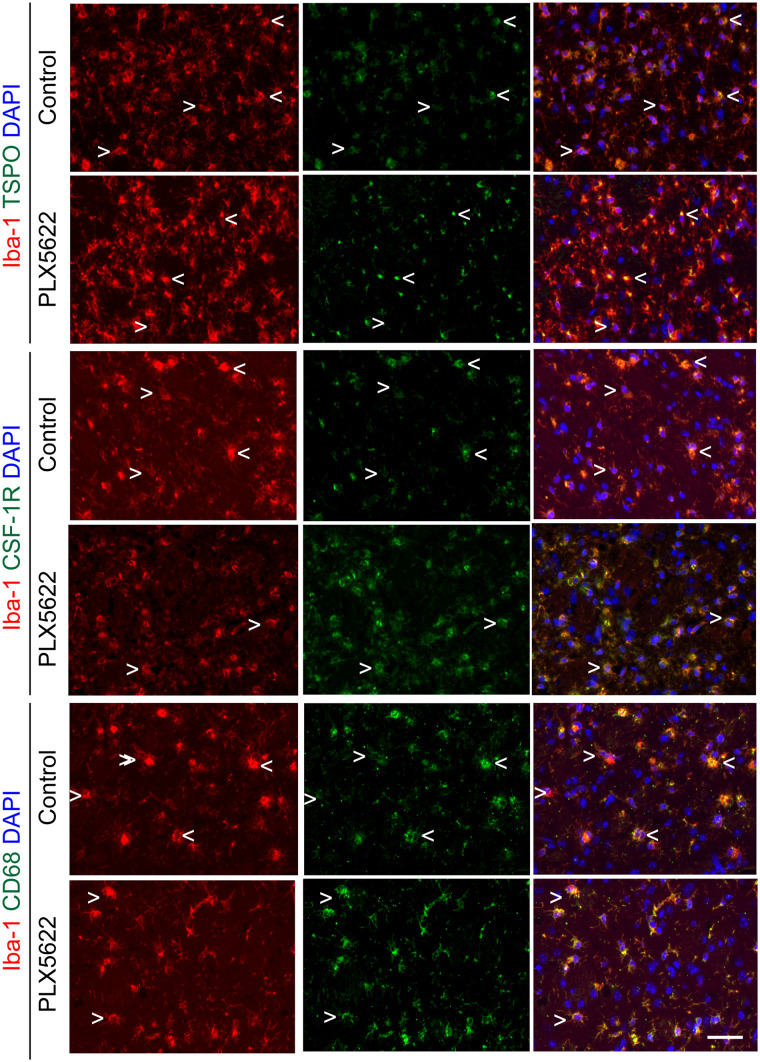
Immunofluorescent staining for TSPO, CSF-1R, and CD68 in Iba-1–positive cell population in both control and PLX5622-treated mice on day 14 after ischemia. < = Iba-1–positive cell positive for marker; > = Iba-1–positive cell negative for marker; scale bar = 20 µm. DAPI = 4′,6-diamidino-2-phenylindole.

We further characterized the Iba-1–positive cell population within the infarct by immunofluorescence (Fig. 3). Both control and PLX5622-treated mice showed a mixed population of Iba-1–positive, TSPO–positive, and Iba-1–positive TSPO-negative cells within the infarct, whereas TSPO expression was higher in PLX5622-treated mice, in line with the ^18^F-DPA-714 PET data acquired on day 14. However, PLX5622-treated mice showed a higher number of Iba-1–positive CSF-1R–positive, and Iba-1–positive CD68–positive (activated myeloid cells) cells within the infarct than did control mice, in line with the repopulation process.

We investigated the potential treatment effect of short-term CSF-1R inhibition on (GFAP-positive) astrocytes on days 14 and 35 after ischemia (Supplemental Fig.7). On day 14, PLX5622-treated mice showed a significantly higher percentage of GFAP-positive area than did control mice within the infarct (*P* = 0.014) or the contralateral striatum (*P* < 0.001). A sustained effect was observed in the contralateral striatum on day 35. However, no colocalization between GFAP and TSPO was observed, indicating that astrocytes did not contribute to ^18^F-DPA-714 PET signal.

**FIGURE 4.**
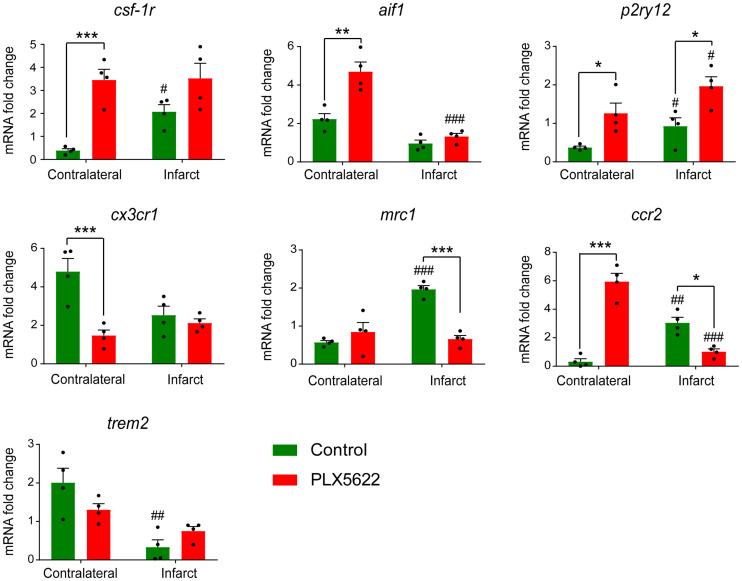
Gene expression of microglia- or macrophage-related markers on day 14 after ischemia. Treatment: *P < 0.05, **P < 0.01, ***P < 0.005. Contralateral hemisphere: ^#^*P* < 0.05, ^##^*P* < 0.01, ^###^*P* < 0.005. mRNA = messenger RNA.

Gene expression of microglia- or macrophage-related markers was assessed on day 14 (Fig. 4). PLX5622-treated mice showed significantly higher *csf-1r* (*P* = 0.001) and *aif1* (also known as Iba-1) (*P* = 0.008) expression than did control mice in the contralateral hemisphere. A good correlation between *csf-1r* and *aif1* was observed (*R*^2^ = 0.86, *P* = 0.34, Supplemental Fig. 8). Moreover, PLX5622-treated mice showed a significant increase in *p2ry12* expression, a marker for antiinflammatory activated microglia, compared with control mice in both infarct (*P* = 0.043) and contralateral (*P* = 0.01) hemispheres. On the other hand, PLX5622 treatment significantly decreased *cx3cr1* (fractalkine receptor constitutively present on microglia and macrophages, *P* = 0.009) in the contralateral hemisphere and *mrc1* (mannose receptor expressed by central nervous system macrophages, *P* = 0.009) expression in the infarct hemisphere. Expression of *ccr2,* found mostly on monocytes, was significantly increased in the contralateral hemisphere (*P* = 0.002) but decreased in the ipsilateral hemisphere (*P* = 0.048) (Fig. 4). No treatment effect was observed on *trem2* expression, a marker of phagocytic activity (*P* > 0.05). Gene expression analysis on day 35 after ischemia indicated long-lasting treatment effects on *aif1, cx3cr1,* and *trem2* gene expression (Supplemental Fig. 9).

**FIGURE 5.**
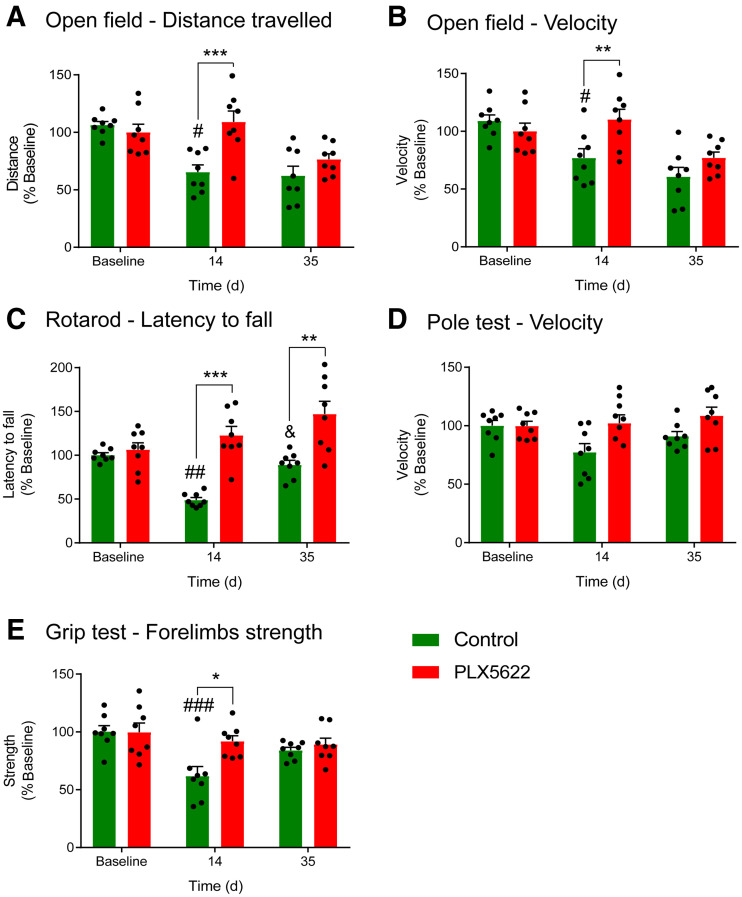
Behavioral tests: distance traveled (A), speed in open field (B), latency to fall during rotarod test (C), velocity during pole test (D), and forelimb strength as assessed with grip test (E). Treatment: *P < 0.05, **P < 0.01, ***P < 0.005. Baseline: ^#^*P* < 0.05, ^##^*P* < 0.01, ^###^*P* < 0.005. Day 14: ^&^*P* < 0.05.

Behavioral tests indicated that PLX5622-treated mice traveled a longer distance (*P* < 0.001) and more quickly on day 14 (*P* = 0.009) than did control mice in the OF (Figs. 5A and 5B). A treatment effect was also observed on the latency to fall in the rotarod test, with PLX5622-treated mice having an increased latency to fall compared with control mice (*P* < 0.005) on both day 14 and day 35 after ischemia (Fig. 5C). No treatment effect was observed on motor functions or coordination assessed with the pole test (*P* = 0.47) (Fig. 5D). Additionally, forelimb strength was significantly better in PLX5622-treated mice on day 14 (*P* = 0.038) (Fig. 5E). No correlation between behavioral parameters and tracer uptake on day 14 was observed (OF *R*^2^ = 0.01 [*P* = 0.34], rotarod *R*^2^ = 0.1 [*P* = 0.44], grip *R*^2^ = 0.55 [*P* = 0.11]).

## DISCUSSION

In this study, we investigated for the first time (to our knowledge) the immunomodulatory effect of CSF-1R inhibition–induced microglia repopulation in the subacute ischemic period using in vivo multimodal imaging. We demonstrated that ^18^F-DPA-714 PET imaging serves as a sensitive biomarker of glial repopulation. In both experimental groups, the Iba-1–positive cell population was one of the major sources of TSPO expression whereas astrocytes did not express TSPO. After 1 wk of repopulation, the Iba-1–positive cells displayed significantly higher expression of TSPO, CSF-1R, and CD68, with altered morphology at the periphery of the infarct and in the contralateral striatum, indicative of an activated state. Gene expression analysis indicated a treatment effect on microglia- and macrophage-related gene expression in line with the repopulation process. *Csf-1r* and *p2ry12* expression increased during repopulation, and increased *csf-1r* expression positively correlated with *aif1* levels, indicative of microglia and macrophage repopulation with a potential switch toward an antiinflammatory state. Gene expression of peripheral cell recruitment markers was altered during repopulation, indicating increased recruitment of peripheral immune cells during microglia depletion whereas repopulation may reverse the effect. This hypothesis was supported by the significantly increased spleen uptake after depletion, as a sign of increased systemic inflammation, and by the subsequent decrease during the repopulation process in PLX5622-treated mice. Additionally, functional outcomes were improved with repopulation: PLX5622-treated mice showed faster motor recovery on day 14 after ischemia, including a longer distance traveled and a higher velocity in 3 of the 4 selected tests, suggesting that repopulation could rescue motor functions. Altogether, this study highlighted the renewal of the microglia and macrophage compartment with CSF-1R inhibitor as a possible new immunomodulatory treatment paradigm after stroke that might be tracked by ^18^F-DPA-714 PET imaging.

TSPO is a transmembrane protein found at the outer mitochondrial membrane involved in metabolic functions (*16*). TSPO expression is markedly increased in glial cells (microglia and astrocytes) and peripheral immune cells (monocytes and lymphocytes) under inflammatory conditions, making it a suitable biomarker of inflammation or activated immune cells (*17*).

^18^F-DPA-714 PET has been used in many translational studies to track TSPO-dependent inflammatory reactions after stroke (*7**,**9**,**10**,**15*). Many studies have confirmed that the dynamics of ^18^F-DPA-714 tracer correlate with CD11b-positive TSPO-positive or Iba-1–positive TSPO-positive cell number, supporting its use for in vivo tracking of microglia- and macrophage-targeting immunomodulatory therapies. However, TSPO PET imaging alone may not be suitable to determine a treatment time window or assess the therapeutic outcome. The main reason is that TSPO has multiple cellular sources, which show a continuum of differentiated states going from pro- to antiinflammatory phenotypes. Therefore, interpretation of the TSPO PET signal benefits from dedicated ex vivo analyses for characterization of the inflammatory response. Nevertheless, in vivo PET imaging may support the determination of therapeutic windows of immunomodulatory compounds by allowing intraindividual follow-up and visualization of disease-relevant molecular processes.

This study supports the use of ^18^F-DPA-714 to track microglia/macrophage repopulation after CSF-1R inhibitor withdrawal. We observed an increased TSPO PET signal within the infarct on days 14 and 21, cross-validated by immunohistochemistry or fluorescence. Dual labeling indicated Iba-1–positive cells as one of the major cellular sources of TSPO expression, whereas astrocytes did not show any TSPO expression, indicating that uptake was modulated mostly by the microglia/macrophage activity.

On the basis of previously published data (*7*), a partial depletion can be expected. According to previous studies, CSF-1R inhibition triggers increased peripheral immune cell infiltration (*4**,**5*). Altogether, it could explain the similar uptake at the end of the treatment period (day 7). Additionally, a recent report indicated that the repopulation kinetic depends on the extent of microglia depletion (*18*). Partial depletion resulted in a slower repopulation rate than did full depletion. Microglia numbers did not recover within the first week after partial depletion, whereas the numbers exceeded control level by +160% after complete depletion (*19*). This effect may partly explain the significantly increased TSPO expression on days 14 and 21 after ischemia. Expression of many microglia-related markers was increased on day 14 after ischemia in the infarct or contralateral hemisphere, in line with the repopulation process.

The CSF-1R/CSF-1 axis regulates developmental functions such as proliferation and survival of microglia (*20*). Here, our data indicated a global significant increase in *csf-1r* and CSF-1R expression in PLX5622-treated mice on day 14 after ischemia, compared with control animals, as a sign of microglia repopulation after inhibitor withdrawal. Additionally, P2ry12 is a specific marker for microglia that is downregulated in a proinflammatory environment and upregulated with exposure to antiinflammatory stimuli, turning into an interesting biomarker for antiinflammatory microglia (*21*). Our data indicated a flare effect on *p2ry12* expression on day 14 after ischemia, suggesting that repopulation may trigger a beneficial antiinflammatory state favoring tissue recovery. Further characterization indicated the presence of a highly activated Iba-1–positive CD68–positive cell population in PLX5622-treated mice triggered by the repopulation process, as previously reported (*18*).

Although gene expression analysis indicated increased microglia-related genes such as *csf-1r, p2ry12,* and *aif1*, peripheral immune cell–related genes such as *ccr2* and *cx3cr1* were significantly downregulated during repopulation within the infarct. Therefore, we hypothesized that subacute CSF-1R inhibition triggered peripheral recruitment and that repopulation reversed the process. Supporting this hypothesis, we observed significantly increased ^18^F-DPA-714 uptake in the spleen during depletion, suggesting an increased adaptive or peripheral inflammatory response with treatment. However, uptake in peripheral organs must be corrected for metabolites for exact interpretation. Still, we supported previous studies showing that CSF-1R inhibition could trigger the engraftment of peripherally derived macrophages into parenchyma and that repopulation reduced gene expression involved in monocyte chemoattraction and leukocyte transmigration (*3**,**4**,**22*).

In our model of mild cerebral ischemia, repopulation accelerated motor function recovery as shown in 3 of the 4 behavioral tests. Behavioral parameters did not correlate with the TSPO PET signal assessed on day 14. We hypothesized that changes in the immune cell population could partly explain the faster recovery in PLX5622-treated mice, including renewal of the microglia compartment or an increased antiinflammatory central or peripheral cell population within the infarct. A few studies reported that short-term depletion after a brain lesion promoted functional recovery (*3**,**23*), correlating with changes in neuroprotective factor expression.

Late invasive data indicated that repopulation reduced the number of Iba-1–positive cells within the infarct, decreased *cx3cr1* expression and *trem2*-dependent phagocytic activity on day 35 after ischemia, but increased astrogliosis. This result suggests that repopulation may resolve the stroke-induced microglia and macrophage phenotype.

## CONCLUSION

We demonstrated that microglia/macrophage repopulation after short-term CSF-1R inhibition could be assessed by in vivo ^18^F-DPA-714 PET/CT and MR imaging. Repopulation induced changes in glial morphology, phenotype, gene expression, and cell recruitment, with signs of improved functional recovery. Further evaluations should identify which cell subpopulations are responsive to CSF-1R inhibition and repopulation. Overall, we propose a promising immunotherapy paradigm targeting microglia activity potentiating stroke recovery.

## Notes

KEY POINTS **QUESTION:** Is PET imaging a valuable tool to assess the immunomodulatory effect of microglia-targeting therapy? **PERTINENT FINDINGS:** Brain repopulation in the inflammatory environment showed therapeutic effects on stroke outcomes. **IMPLICATIONS FOR PATIENT CARE:** ^18^F-DPA-714 PET imaging allows tracking of the repopulation process after short-term CSF-1R inhibition.
